# *Staphylococcus aureus* RNAIII Binds to Two Distant Regions of *coa* mRNA to Arrest Translation and Promote mRNA Degradation

**DOI:** 10.1371/journal.ppat.1000809

**Published:** 2010-03-12

**Authors:** Clément Chevalier, Sandrine Boisset, Cédric Romilly, Benoit Masquida, Pierre Fechter, Thomas Geissmann, François Vandenesch, Pascale Romby

**Affiliations:** 1 Architecture et Réactivité de l'ARN, Université de Strasbourg, CNRS, IBMC, Strasbourg, France; 2 INSERM U851, Centre National de Référence des Staphylocoques, Lyon, France; Université de Lyon, Lyon, France; Dartmouth Medical School, United States of America

## Abstract

*Staphylococcus aureus* RNAIII is the intracellular effector of the quorum sensing system that temporally controls a large number of virulence factors including exoproteins and cell-wall-associated proteins. Staphylocoagulase is one major virulence factor, which promotes clotting of human plasma. Like the major cell surface protein A, the expression of staphylocoagulase is strongly repressed by the quorum sensing system at the post-exponential growth phase. Here we used a combination of approaches *in vivo* and *in vitro* to analyze the mechanism used by RNAIII to regulate the expression of staphylocoagulase. Our data show that RNAIII represses the synthesis of the protein through a direct binding with the mRNA. Structure mapping shows that two distant regions of RNAIII interact with *coa* mRNA and that the mRNA harbors a conserved signature as found in other RNAIII-target mRNAs. The resulting complex is composed of an imperfect duplex masking the Shine-Dalgarno sequence of *coa* mRNA and of a loop-loop interaction occurring downstream in the coding region. The imperfect duplex is sufficient to prevent the formation of the ribosomal initiation complex and to repress the expression of a reporter gene *in vivo*. In addition, the double-strand-specific endoribonuclease III cleaves the two regions of the mRNA bound to RNAIII that may contribute to the degradation of the repressed mRNA. This study validates another direct target of RNAIII that plays a role in virulence. It also illustrates the diversity of RNAIII-mRNA topologies and how these multiple RNAIII-mRNA interactions would mediate virulence regulation.

## Author Summary

*Staphylococcus aureus* causes a wide spectrum of diseases in humans and is one of the main causes of community- as well as hospital-acquired infections. *S. aureus* produces a large number of virulence factors that are expressed in a coordinated manner and at appropriate time and space. To this end, a set of multiple *trans*-acting regulators, including regulatory proteins and RNA, is brought into play. The ability of organisms to use RNA to modulate gene expression is a relatively new concept. This is the case for the largest regulatory RNA, *S. aureus* RNAIII, which controls the switch between the expression of surface proteins and excreted toxins. Here we used a combination of approaches *in vivo* and *in vitro* to analyze the mechanism used by RNAIII to regulate the expression of one major virulence factor, staphylocoagulase, which promotes clotting of human plasma. We found that RNAIII regulates the expression of staphylocoagulase through direct interactions with its mRNA. RNAIII binds to two distant regions of *coa* mRNA to arrest translation and in a coordinated manner, the endoribonuclease III recognizes the formed duplex to initiate degradation of the repressed mRNA. Staphylocoagulase belongs to the early expressed virulence factors, such as protein A, that are repressed by RNAIII using a similar dual mechanism. This study illustrates the diversity of RNAIII-mRNA topologies and how these multiple RNAIII-mRNA interactions would mediate virulence regulation.

## Introduction

*Staphylococcus aureus* is ubiquitous in the environment and is a commensal organism found on human skin. This major human pathogen is the most common cause of hospital- and community-acquired infections. Therefore, *S. aureus* has developed a plethora of strategies to survive in various environmental niches. The broad range of human infections caused by *S. aureus* is in part due to the production of a large number of virulence factors. These factors mediate cell and tissue adhesion, contribute to tissue damage and spreading, and protect the bacteria against the host immune defense system. Coordinated virulence gene expression is thought to be critical for infection and is orchestrated by multiple factors involving two-component systems, global regulatory proteins, and the quorum-sensing system [1],[2]. Quorum-sensing regulation in staphylococci is mainly driven by the *agr* system, which was shown to exert a variety of functions in bacterial physiology and pathogenesis [2],[3]. The *agr* system is composed of two divergent transcription units, RNAII and RNAIII. RNAII contains a density-sensing cassette (*agr*D and B) and a two-component sensory transduction system (*agr*A and C). Upon a threshold level of cell density, the response regulatory protein, AgrA, activates the transcription of its own operon and of the regulatory RNAIII [4]. Recent data show the existence of two distinct *agr* regulatory circuits; one is RNAIII-independent and the other is RNAIII-dependent [3]. Although RNAIII controls the expression of many virulence factors, the expression of several enzymes involved in carbohydrate and amino acid metabolisms are downregulated by an unknown mechanism that is independent of RNAIII. Furthermore, AgrA directly activates the synthesis of several phenol-soluble modulin (PSM) peptides at the transcriptional level [3]. Hence, AgrA and RNAIII act in concert to regulate the synthesis of many proteins in response to cell density, interconnecting metabolism, and virulence gene expression [3],[5],[6].

RNAIII has a dual function because it acts as a mRNA that encodes a PSM peptide, Δ-hemolysin, and temporally controls the switch between early expression of surface proteins and late expression of several exotoxins [1]. RNAIII belongs to the class of *trans-*acting RNAs, which regulate several mRNAs at the post-transcriptional level [7],[8]. The 5′ domain of RNAIII activates translation of *hla* mRNA (encoding α-hemolysin) by preventing the formation of an intramolecular mRNA structure that sequesters the *hla* ribosome binding site [1],[9]. The 3′ end and the central domain of RNAIII (Fig. S1) repress the synthesis of early expressed cell surface virulence factors (protein A, fibrinogen-binding protein) as well as the transcriptional regulator, Rot, the repressor of toxins [1], [10]–[12]. We have previously shown that the 3′ domain of RNAIII, which is the most highly conserved domain, could also form base pairings with *coa* mRNA encoding staphylocoagulase [12]. Staphylocoagulase is an extracellular protein produced by almost all clinical isolates of *S. aureus*, which specifically forms a complex with prothrombin, the so-called staphylothrombin, to promote fibrin formation in human plasma. Like the major cell surface protein A, the synthesis of staphylocoagulase is growth-phase dependent, and the protein is produced during exponential growth and rapidly repressed by the *agr* system [13].

We show here that RNAIII is responsible for the *in vivo* repression of staphylocoagulase at the post-transcriptional level. This results from a direct interaction of two distant domains of RNAIII with *coa* mRNA. The complex is formed rapidly and is stable enough to prevent the binding of the ribosomal 30S subunit and, in addition, provides binding sites for the endoribonuclease III. Thus, *coa* mRNA belongs to the RNAIII-dependent repressed mRNAs that are regulated by a similar mechanism. This work and previous data also illustrate the variety of RNAIII-mRNA topologies that are sufficient to block the access of the ribosome at the initiation step.

## Results

### RNAIII regulates the synthesis of staphylocoagulase at the post-transcriptional level

**Figure 1:**
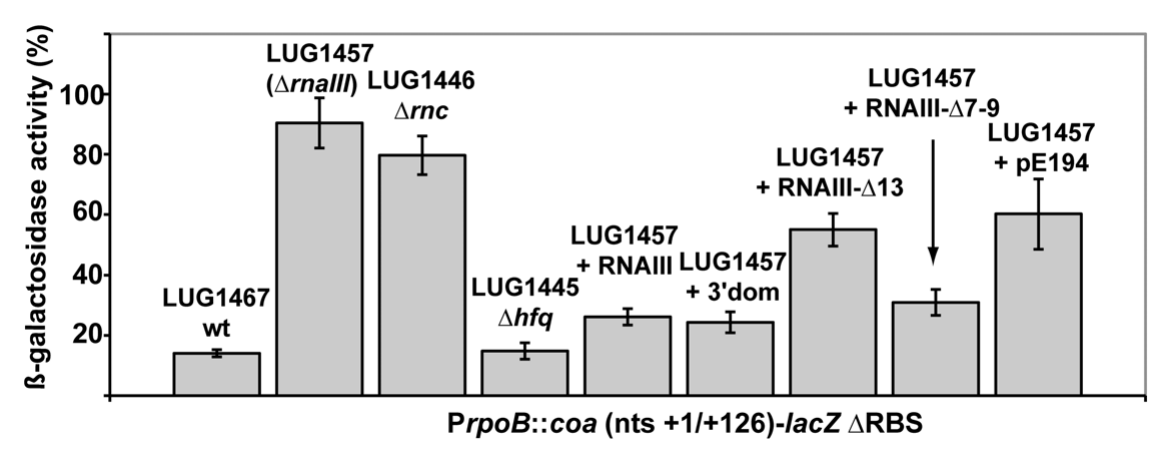
RNAIII-dependant regulation of *coa* mRNA *in vivo*. β-galactosidase activity detected from different gene fusions. β-galactosidase activity measured from P*rpoB-coa (+1/+126)*::*lacZ* fusions in various *S. aureus* strains: LUG1467 (*rnaIII+*, wt), LUG1446-Δ*rnc* (deletion of *rnc* gene encoding RNase III), LUG1445-Δ*hfq* (deletion of *hfq* gene), LUG1457 (Δ*rnaIII*), and LUG1457 transformed with the plasmid expressing the wild type RNAIII, the 3′ domain, RNAIII-Δ13 (RNAIII deleted of hairpin 13), or RNAIII-Δ7–9 (RNAIII deleted of hairpins 7 to 9), or with the plasmid containing no insert (pE194). The β-galactosidase activity was normalized for total cell density and is represented as a percentage of the uninhibited control (LUG1457). The results represented a mean of three independent experiments.

Sequence complementarity between RNAIII [nucleotides (nts) 391 to 437] and *coa* mRNA (nucleotides 15 to 52) suggested that the 3′ domain of RNAIII can repress *coa* expression at the post-transcriptional level through the formation of RNAIII-mRNA interactions [12]. To validate the *in vivo* relevance of such a mechanism, we analyzed the expression of gene reporter constructs in various *S. aureus* strains that expressed the wild type RNAIII or truncated versions of RNAIII. The 5′ start of *coa* mRNA was determined by 5′ rapid amplification of cDNA ends (RACE) showing that the 5′ untranslated region contains 35 nucleotides upstream the AUG initiation codon. The entire leader regulatory region of the *coa* gene, including 88 nucleotides of the coding sequence, was cloned in-frame with the *lacZ* gene into the pTCV-*lac* shuttle vector [14]. This construct is under control of an *agr*-independent promoter (P*rpo*B). The β-galactosidase activity was determined in the *S. aureus* strain LUG1467 (wt, *rnaIII*+), which express RNAIII and in LUG1457 (Δ*rnaIII*), which carries a deletion of the *rnaIII* gene (Fig. 1A). We also measured the synthesis of the β-galactosidase from the *coa*-*lacZ* fusion in the strains lacking the *rnc* gene, encoding the endoribonuclease III (RNase III) (LUG1446, Δ*rnc*), or of *hfq* gene, encoding the Sm-like Hfq protein (LUG1445, Δ*hfq*). The β-galactosidase activity was reduced six-fold in the LUG1467 strain (wt, *rnaIII* ^+^) compared to the LUG1457 (Δ*rnaIII*) strain. Furthermore, Hfq had no significant effect on the RNAIII-dependent repression, while the deletion of *rnc* alleviated the repression of the *coa*-*lacZ* fusion (Fig. 1A).

Experiments were also carried out on the LUG1457 strain (Δ*rnaIII*), complemented with different sets of plasmid pLUG274 expressing either the wild-type RNAIII, the 3′ end domain comprising nts 391 to 516, RNAIII-Δ13 (deletion of hairpin 13) or RNAIII-Δ7-9 (deletion of hairpins 7 to 9) (Fig. 1A). A control experiment was carried out with the plasmid pE194 with no insert. Unexpectedly, this plasmid slightly decreased the β-galactosidase levels compared to the LUG1457 (Δ*rnaIII*) strain. One explanation would be that the copy number of the pTCV-*lac* derivative was affected by the presence of the multicopy plasmid, pE194, even though both plasmids are compatible. However, derivatives of plasmid pE194 producing high levels of wild-type RNAIII, the 3′ domain, or RNAIII-Δ7-9 reproducibly decreased the synthesis of β-galactosidase (Fig. 1A). Conversely, the expression of RNAIII-Δ13, which lacks the base-pairing complementarities with *coa* mRNA, did not alter β-galactosidase synthesis (Fig. 1A).

We also analyzed the steady-state level of *coa* mRNA in different *S. aureus* strains in late-exponential phase (Fig. S2). The mRNA was not detected in RN6390 (wt, *rnaIII* ^+^) while its level was significantly enhanced in the isogenic strain lacking *rnaIII* gene (Δ*rnaIII*). Of interest, in the Δ*rnc* strain, the level of *coa* mRNA was reproducibly found to be slightly higher than in the parental wt strain (Fig. S2). This result suggests that the RNase III-dependent degradation of the mRNA contributes in part to the disappearance of the mRNA pool. Complementation assays were also done with the mutant Δ*rnaIII* strain transformed with plasmids expressing several variants of RNAIII. The expression of the 3′ domain of RNAIII strongly reduced the level of *coa* mRNA while significant levels of the mRNA were still detected in the strain expressing RNAIII-Δ13 (Fig. S2).

Taken together, these results strongly suggest that RNAIII and RNase III coordinately repress *coa* expression at the post-transcriptional level and that the hairpin 13 of RNAIII is essential for the repression.

### RNAIII binds to two distant regions of *coa* mRNA

**Figure 2:**
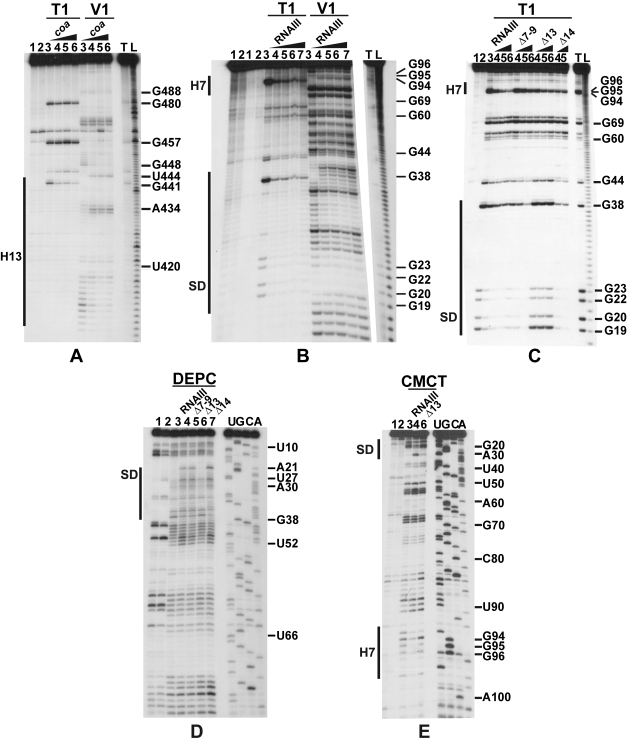
Enzymatic and chemical probing of the structure of the inhibitory RNAIII-*coa* mRNA complex. (A) Enzymatic hydrolysis of 5′-end-labeled 3′ domain of RNAIII, alone (lane 3) or in the presence of an excess of *coa* mRNA (lane 4, 20 nM; lane 5, 50 nM; lane 6, 100 nM; lane 7, 250 nM). Lanes 1, 2: incubation controls on free RNA or bound to *coa* mRNA, respectively. Lanes T, L: RNase T1 under denaturing conditions and alkaline ladders, respectively. T1, V1: RNase T1 and RNase V1 hydrolysis, respectively. (B, C) Enzymatic hydrolysis of 5′-end-labeled *coa* mRNA, alone (lane 3) or bound to the wild type RNAIII, or to the mutant RNAIII deleted of hairpins 7 to 9 (Δ7–9), of hairpin 13 (Δ13) or of hairpin 14 (Δ14). Concentrations of wild type or mutant RNAIII: lane 4, 20 nM; lane 5, 50 nM; lane 6, 100 nM; lane 7, 250 nM. Same legend as in A. (D) DEPC (N7A) modification of unlabeled *coa* mRNA, free (lane 3) or bound to the wild type RNAIII (lane 4), or the mutant RNAIII deleted of hairpins 7 to 9 (Δ7–9, lane 5), of hairpin 13 (Δ13, lane 6) or of hairpin 14 (Δ14, lane 7) at 200 nM. Lanes U, G, C, A: dideoxy-sequencing reactions performed on *coa* mRNA. (E) CMCT modification of unlabeled *coa* mRNA. Same legend as in D. Reactivity changes are indicated by bars on one side of each autoradiography. SD is for Shine-Dalgarno sequence and H7 is for hairpin 7 of RNAIII.

**Figure 3:**
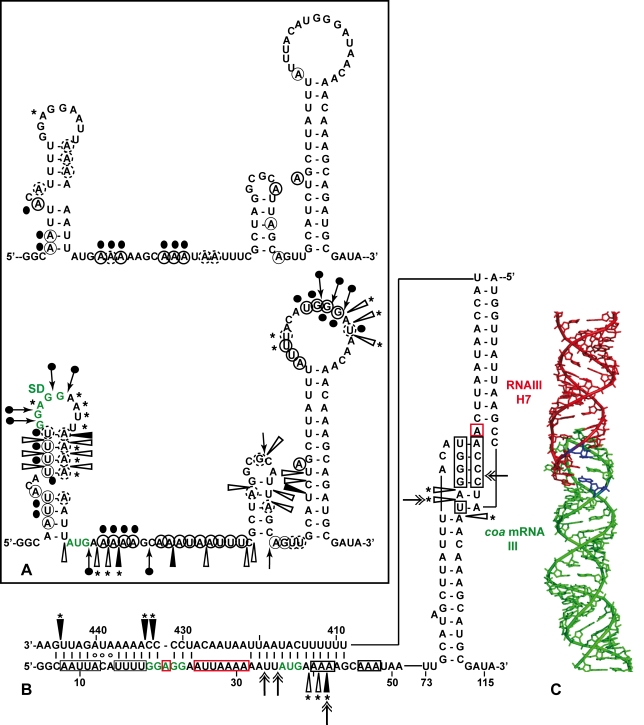
Structure of the RNAIII-*coa* mRNA complex. (A) Summary of the enzymatic cleavages and chemical reactivities of nucleotides of *coa* mRNA. Enzymatic cleavages are given as follows: RNase T1 (black arrow), and RNase V1 (white arrowhead) moderate, (black arrowhead) strong cleavage. Chemical modifications of cytosines at N3, and adenines at N1 by DMS, of uridines at N3 and guanines at N1 by CMCT, and of adenines at N7 by DEPC: full and dashed circled nucleotides are for strong and moderate reactivity, respectively. No symbol is for non reactive, nd is for not determined due to non-specific cleavages or pauses of RT in the incubation control. The reactivity of A at N7 is reported on the secondary structure shown in the insert. Reactivity changes induced by the binding of RNAIII are indicated as follows: black circles denote strong protection, enhancements and new RNase V1 cleavages are represented by asterisks. (B) Secondary structure model of the RNAIII-*coa* mRNA complex showing the reactivity changes induced by complex formation: RNase V1 (white and black arrowheads), RNase III cleavages (double arrow). Effect of RNAIII binding: Protected nucleotides are squared in black and the nucleotides, which become accessible, are squared in red. (C) The topology of the loop-loop interaction built by graphic modeling based on the probing data. The hairpin III of *coa* mRNA is in green and the hairpin 7 of RNAIII in red. The RNase III cleavages are shown in blue.

The predicted base-pairing between RNAIII and *coa* mRNA and the *in vivo* experiments suggested that the RNAIII-dependent repression of *coa* mRNA was governed by direct RNAIII-mRNA pairing. We thus mapped the regions of interactions using enzymatic and chemical probing. The conformation of *coa* mRNA was probed using RNase T1 (specific for unpaired guanines), RNase V1 (specific for helical regions), and several base-specific chemicals such as dimethylsufate (methylates N1A≫N3C), a carbodiimide derivative (modifies N3U≫N1G), and diethylpyrocarbonate (carboxyethylates N7A). Several experiments of RNA structure probing are shown in Fig. 2. The secondary structure model of *coa* mRNA, which explains most of the probing data, is comprised of three stem-loop structures connected by unpaired residues (Fig. 3A). The AU-rich hairpin I is of weak stability but is proposed to occur based on the enzymatic cleavage pattern. However, the coexistence of alternative structures in the region encompassing nucleotides 10 to 70 may explain the concomitant presence of RNase V1 cleavages and the reactivity of many nucleotides at one of their Watson-Crick positions. In contrast, the long hairpin structure III located in the coding region of *coa* mRNA is well supported by the enzymatic cleavage patterns and the non reactivity of the Watson-Crick position of A77 to U85 towards chemicals (Fig. 2B-E). Binding of RNAIII induced changes in the region encompassing the ribosome binding site (RBS, nucleotides U10 to A48). RNAIII protected the guanines of the Shine-Dalgarno (SD) sequence against RNase T1 as well as the nucleotides U10 to U18 and A40 to A48 against chemical modifications (Fig. 2D, E). Concomitantly, RNAIII binding induced new RNase V1 cleavages at positions 39–41 and enhanced reactivity of A21, A24, A25, A29 to A31 at position N1, of A21 at position N7, and of U26 and U27 at position N3 in *coa* mRNA (Fig. 2D–E, Fig. 3B). These reactivity changes in the RBS of *coa* mRNA most likely resulted from the binding of the hairpin 13 of RNAIII because its deletion in RNAIII conferred no additional effect on the accessibility of the RBS of *coa* mRNA (Fig. 2C). Binding of *coa* mRNA to the 3′ domain or to RNAIII induced correlated changes in hairpin 13. Strong protections were observed at G441 against RNase T1 and at positions 411–415 and 448–449 against RNase V1 (Fig. 2A). Concomitantly, increased RNase V1 cleavages were observed at positions 433–434 and 444. All these data are thus consistent with the formation of a RNAIII-mRNA duplex that sequestered the RBS of *coa* mRNA (Fig. 3). This imperfect duplex involves two consecutive regions of 13 base-pairings interrupted by an internal loop and a bulged adenine 21 (Fig. 3B).

Unexpectedly, we also found a second RNAIII binding site restricted to the apical loop III of *coa* mRNA (Fig. 3B). Binding of RNAIII reduced considerably the RNase T1 cuts at G94-97 and the modifications of the nucleotides UGGGAU98 mediated by the chemicals (Fig. 2B, E). Concomitantly, RNAIII binding induced several RNase V1 cuts at positions 96 to 98 (Fig. 2B). These changes were abolished if the complex was formed between *coa* mRNA and the RNAIII deleted of hairpins 7 to 9 (Fig. 2C). Furthermore, *coa* mRNA binding to RNAIII reduced significantly the reactivity of the nucleotides CCCA243 towards DMS in the apical loop 7 of RNAIII. These data are strengthened by the sequence complementarities between the apical loop III of *coa* mRNA and the hairpin loop 7 of RNAIII and support the existence of a loop-loop interaction (Fig. 3A, B). This interaction is, however, strongly dependent on the formation of the imperfect duplex because the reactivity changes in the hairpin loop III of *coa* mRNA were significantly decreased if complex formation was performed with RNAIII-Δ13 (Fig. 2C). Molecular modeling of the RNA interaction between the two loops shows an almost continuous stacking from the 3′ side of the helix III of *coa* mRNA, through the loop-loop intermolecular helix to the helix of the hairpin 7 of RNAIII. The two connecting loops of three and two nucleotides bridge the grooves of the newly formed helix (Fig. 3C).

Altogether, the data show that the mRNA-RNAIII complex is composed of a bipartite site, which implies the formation of an imperfect duplex and a loop-loop interaction.

### The ribosome binding site of *coa* mRNA is the major recognition site for RNAIII

**Figure 4:**
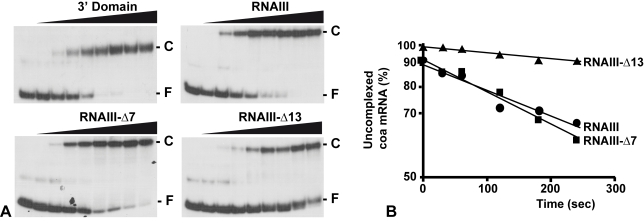
RNAIII binds efficiently to *coa* mRNA *in vitro*. (A) Determination of the apparent dissociation constant for RNAIII-*coa* mRNA complex. 5′-end-labeled *coa* mRNA was incubated alone (−) or with various concentrations of unlabeled wild type RNAIII, the 3′ domain, RNAIII-Δ7-9, and RNAIII-Δ13 (1, 5, 10, 20, 50, 100, 200, and 250 nM). The fraction of labeled *coa* mRNA associated with RNAIII or its derivatives was calculated from the counts in the corresponding band relative to the total counts in the lane. The Kd value was estimated as the concentration of RNAIII allowing 50% of *coa* mRNA binding. (B) Binding rate constant for various RNAIII-*coa* mRNA complexes as determined from three independent experiments. 5′-end-labeled *coa* mRNA (0.1 nM) was incubated with unlabeled RNAIII (20 nM), RNAIII-Δ7-9 (20 nM), and RNAIII-Δ13 (20 nM) at 37°C. Aliquots were withdrawn at various times (from 0 to 350 sec). The percentage of free *coa* mRNA was plotted as a function of time to estimate the association rate constant according to [40]. The values for the binding rate constants are the means of three independent experiments: 1.1×10^5^ M^−1^ s^−1^ (RNAIII), 9.5×10^5^ M^−1^ s^−1^ (RNAIII-Δ7-9), and 1.1×10^4^ M^−1^ s^−1^ (RNAIII-Δ13).

The contribution of the two binding sites toward complex formation was further evaluated by gel shift assays. Each experiment has been reproduced four times. *In vitro* labeled *coa* mRNA was first incubated with increasing concentrations of RNAIII or its variants (RNAIII-Δ13, RNAIII-Δ7–9, and the 3′ domain) at 37°C for 15 min (Fig. 4A). This experiment shows that *coa* mRNA binds to RNAIII or its 3′ domain with a K_d_ value of around 10 nM. The deletion of hairpins 7 to 9 in RNAIII had only a two-fold effect on the dissociation constant (around 25 nM), while the deletion of hairpin 13 in RNAIII increased significantly the Kd value by one order of magnitude (around 150 nM).

The initial rate of wild type RNAIII binding to 5′ end-labeled *coa* mRNA was estimated from a time-course analysis and resulted in an association rate constant of 1.1×10^5^ M^−1^ s^−1^ (Fig. 4B). Similar values were observed for three other RNAIII-mRNA (*spa*, SA1000, *rot*) target complexes [11],[12]. These data indicate that the complexes are rapidly formed as observed for several fully complementary antisense-target RNA systems [15],[16]. We then investigated whether deletion of hairpin 7 or hairpin 13 of RNAIII involved in the binding would affect binding rates (Fig. 4B). The binding rate constant for the mutant RNAIII-Δ7-9-*coa* mRNA pair was identical to the wild type complex (9.35×10^4^ M^−1^ s^−1^). However, for the mutant RNAIII-Δ13-*coa* mRNA pair, the value was significantly decreased by one order of magnitude lower (1.1×10^4^ M^−1^ s^−1^). These experiments strongly suggest that initial pairings involved the hairpin 13 of RNAIII and that this motif confers stable binding to *coa* mRNA.

### RNAIII binding to *coa* mRNA interferes with the formation of the initiation complex

**Figure 5:**
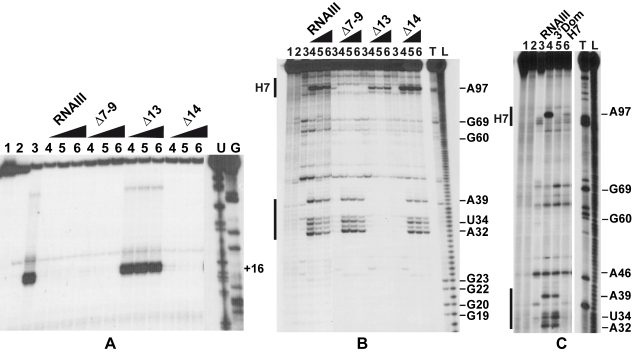
The RNAIII-*coa* mRNA complex prevents ribosome binding and promotes RNase III cleavages. (A) Formation of the ternary complex between *coa* mRNA (15 nM), *S. aureus* 30S ribosomal subunits (250 nM), and initiator tRNA (1 µM) was monitored in the absence (lane 3) or in the presence of increasing concentrations of wild-type RNAIII, RNAIII-Δ7–9 (Δ7–9), RNAIII-Δ13 (Δ13), and RNAIII-Δ14 (Δ14): lane 4, 25 nM; lane 5, 50 nM; lane 6, 100 nM. The toeprint at position +16 is indicated. Lanes 1, 2: Incubation controls on free RNA or RNA bound to RNAIII, respectively. Lanes U, G: dideoxy-sequencing reactions performed on *coa* mRNA. (B) RNase III hydrolysis of 5′-end-labeled *coa* mRNA, alone (lane 3) or in the presence of an excess of wild type RNAIII, RNAIIIΔ7–9 (Δ7–9), RNAIII-Δ13 (Δ13), RNAIII-Δ14 (Δ14): lane 4, 25 nM; lane 5, 50 nM; lane 6, 100 nM. Lanes 1, 2: incubation controls on free *coa* mRNA or bound to RNAIII, respectively. Lanes T1, L: RNase T1 and alkaline ladders, respectively. (C) RNase III hydrolysis of 5′-end-labeled *coa* mRNA, alone (lane 3) or in the presence of an excess of wild type RNAIII (lane 4), the 3′ domain of RNAIII (3′ Dom), and the hairpin 7 of RNAIII (H7). Lanes T1, L: RNase T1 and alkaline ladders, respectively.

Since RNAIII binds to the SD sequence of *coa* mRNA, we analyzed whether RNAIII binding is sufficient to prevent the formation of the ternary initiation complex formed with the *S. aureus* 30S subunit, initiator tRNA^Met^, and *coa* mRNA. Formation of the ternary complex, which blocked the elongation of a cDNA primer by reverse transcriptase, produced a toeprint at U49/A50, 15 nucleotides downstream of the initiation codon ([17]; Fig. 5A). Intriguingly, a second toeprint resulting from ribosome binding was also observed at A14; this weak toeprint was not detected with the *E. coli* ribosomal 30S subunit (result not shown). Binding of RNAIII, RNAIII-Δ7–9 (deleted of hairpins 7 to 9), or RNAIII-Δ14 (deleted of hairpin 14) strongly decreased the two toeprint signals. This indicates that the regulatory RNAIII totally blocks access of the ribosome at the RBS site of *coa* mRNA. The inhibition was observed whether the RNAIII-mRNA complex was pre-formed or RNAIII was added together with the 30S subunit (Fig. 5A). This shows that the resulting inhibitory complex is rapidly formed and sufficiently stable to prevent the formation of the ribosomal initiation complex. Using this assay, we were not able to analyze the contribution of the loop-loop interaction in the inhibition of ribosome binding because the primer used for elongation hybridized in the long hairpin loop III. However, the RNAIII-ΔH13 exerted no inhibitory effect on ribosome binding, showing that the specific RNAIII-mediated inhibition of ribosome binding to *coa* mRNA resulted mainly from the sequestration of the RBS by the hairpin 13 of RNAIII (Fig. 5A).

### RNase III cleaves the two distant mRNA-RNAIII interactions *in vitro*

As RNase III is required for efficient repression *in vivo* (Fig. 1), we analyzed whether this enzyme can cleave the complex *in vitro*. We have shown previously that cleavage assays by RNase III can be a useful tool for probing *in vitro* RNA-RNA complexes [18]. The RNase III-dependent cleavages were probed on the 5′-end labeled RNAs as well as on the native RNAIII-*coa* mRNA complex using a purified His-tagged RNase III from *S. aureus* (Fig. 5B–C). Only weak RNase III cleavages were observed in the free *coa* mRNA. When the 5′ end-labeled mRNA was incubated with RNAIII, four major cleavages occurred at positions 32, 34, 39, and 97 in the mRNA (Fig. 5B–C). Binding of *coa* mRNA induced a RNase III-dependent cleavage at C241 of the labeled RNAIII (results not shown). Thus, the two regions of hybridization were susceptible to RNase III cleavages.

Using truncated versions of RNAIII and the isolated hairpin 7 or the 3′ domain, we were able to assign the partners involved in the RNAIII-mRNA complex. Indeed, the hairpin 7 only induced a specific RNase III-cleavage at position 97 of the mRNA, while the hairpin 13 binding promoted major cleavages at positions 32, 34, and 39 of the mRNA (Fig. 5C). Furthermore, the complex formed between RNAIII-Δ7–9 and *coa* mRNA was cleaved efficiently by RNase III at positions 32, 34, and 39 of the mRNA (Fig. 5B). Conversely, only one RNase III-mediated cleavage was detected at position 97 of *coa* mRNA bound to RNAIII-Δ13 or to hairpin 7 (Fig. 5B–C). This cleavage was, however, weaker than the cleavage found in the wild type complex. These experiments correlate well with the probing data showing that the loop-loop interaction is stabilized by the duplex formed between the RBS of *coa* mRNA and the hairpin 13 of RNAIII. In the irregular duplex, RNase III cleaves only from the mRNA side, whereas the enzyme induces cleavages on both strands of the loop-loop interaction leading to the classical two nucleotides 3′ overhang. Taken together, these data fully support the chemical and enzymatic probing showing that the hairpin 13 of RNAIII binds to the RBS of *coa* mRNA, while the hairpin loop 7 forms limited base pairings with the coding sequence. The data further indicate that the loop-loop interaction adopts a topology that is appropriate for efficient RNase III binding and catalysis [18].

## Discussion

*S. aureus* produces a large variety of virulence factors that are required for the successful colonization of the host and that confer to the bacteria the ability to counteract the immune defense system of the host [2]. Among these virulence factors, staphylocoagulase primarily activates prothrombin, inducing the formation of a fibrin clot around the bacterial cell [19]. Coating the bacteria with host proteins contributes to hiding the bacteria from the immune system and from phagocytosis. The expression of coagulase was shown to follow a temporal regulation, as do several adhesins and surface proteins that are expressed earlier than the secreted enzymes, immunotoxins, and cytotoxins [2]. Furthermore, coagulase belongs to the early expressed virulence factors such as protein A, the fibrinogen-binding protein SA1000, and the SsaA-like protein SA2353, which were found to be repressed by the quorum sensing-controlled RNAIII. During the growth cycle, the level of RNAIII varies inversely with that of *coa* mRNA [13]. In addition, it was shown that the coagulase expression was both positively and negatively controlled by an *agr-* dependent mechanism. A functional *agr* element resulted in a relative elevation of the *coa* mRNA level at the early exponential phase of growth followed by a strong decrease of the mRNA level at the post-exponential phase of growth [13]. We demonstrate here that the *agr*-dependent repression effect on *coa* mRNA is most probably the result of a direct binding of RNAIII to *coa* mRNA.

**Figure 6:**
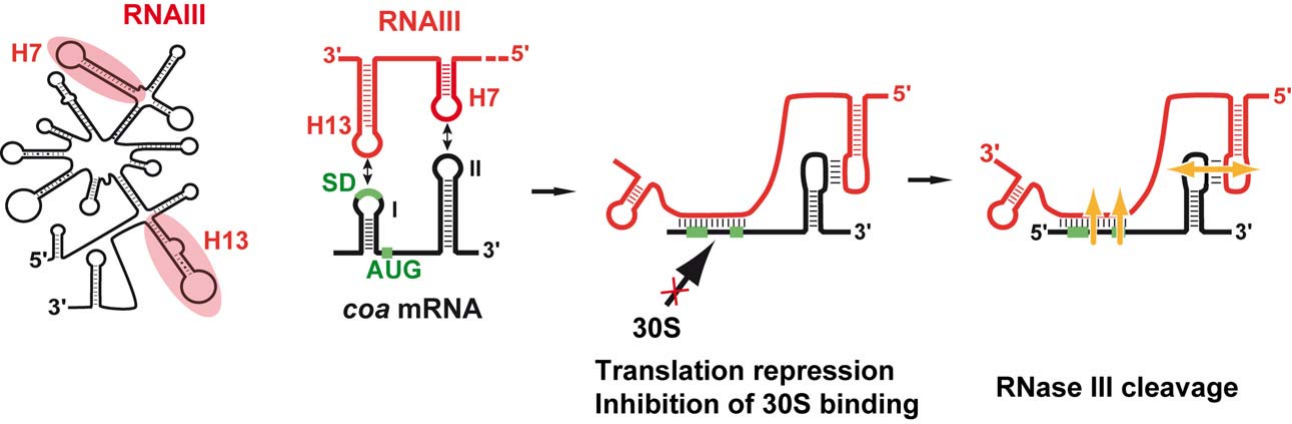
Schematic view of RNAIII-mediated repression of *coa* mRNA. RNAIII binds to its target mRNA masking the RBS and part of the coding sequence. Binding of RNAIII hinders ribosome binding and promotes access to RNase III. SD (Shine-Dalgarno) and AUG are in green. RNAIII is in red, the mRNA target is in black, 30S is for small ribosomal subunit.

We show that RNAIII in conjunction with RNase III are required to fully repress the synthesis of staphylocoagulase at the stationary phase of growth (Fig. 6). The primary effect of RNAIII would be to prevent translation initiation subsequently followed by the RNase III-dependent cleavage of the repressed mRNA. Since we have previously shown that RNase III binds efficiently to RNAIII, we propose that RNAIII-dependent translation repression and RNase III cleavage are coupled. Hence, these data, together with previous works, show that RNAIII represses the synthesis of coagulase, protein A, SA1000, SA2353, and Rot by a similar mechanism [10]–[12]. In addition, probing the mRNA structure also shows that *coa* mRNA adopts a very similar structural organization to *spa* and SA1000 mRNAs (Fig. S3). The three mRNAs have short 5′ untranslated regions, which carry a 5′ hairpin structure with a strong SD sequence located in the apical loop (Fig. S3). In the absence of RNAIII, these elements may confer to the mRNAs a high stability [11]. Indeed, in *B. subtilis*, stabilization of mRNAs was shown to be a consequence of the blocking of the 5′ end by a stalled initiating ribosome at a SD-like sequence [20],[21] or by a stable 5′ hairpin structure and a strong RBS [22]. Therefore, the coordinated action of RNAIII and RNase III would be needed to irreversibly repress the synthesis of these virulence factors at an appropriate time.

*In vitro* binding assays show that RNAIII binds to *coa* mRNA and its other mRNA targets with a rather high association rate constant. Efficient repression by non-coding RNAs (ncRNA), which act at the translational level, requires that the ncRNA binds to target mRNAs within a short time frame, i.e. before the formation of the stable ribosomal initiation complex [15],[16]. Our data also indicate that *S. aureus* Sm-like Hfq protein is not required for the RNAIII-dependent repression of *coa* mRNA *in vivo* (Fig. 1), in contrast to *Escherichia coli* and *Salmonella typhimurium* ncRNAs which act in concert with Hfq to bind mRNA targets [23],[24]. Despite the fact that Hfq binds to RNAIII [11], the observation that the deletion of *hfq* does not exhibit severe phenotypic defects rules out the direct involvement of Hfq in regard to RNAIII-mediated regulation in *S. aureus* [10],[25]. Instead, we propose that the structures of RNAIII and its mRNA targets may compensate for the need of a helper protein as shown for antisense RNAs fully complementary to their target mRNAs [15],[16]. We, however, do not rule out that another protein or RNase III could contribute to stabilize and/or facilitate the formation of the hybrid [11].

The regions of interaction in RNAIII and *coa* mRNA contained stem-loop structures that are indeed well appropriate for initial loop-loop interactions. The two conserved C-rich loops, 7 and 13, of RNAIII bind to the RBS and to the hairpin loop III in the coding sequence of *coa* mRNA, respectively (Fig. 3, 6). These C-rich hairpin loops of RNAIII are also used to repress the other mRNA targets, although the topologies of the resulting inhibitory complexes are different (Fig. S3). RNAIII forms long duplexes with the RBS of *spa* and SA1000 mRNAs, while it forms two loop-loop interactions with the 5′UTR and the RBS of *rot* mRNA, respectively ([11],[12], Fig. S3). Here we show that the RNAIII-*coa* mRNA complex involves an imperfect duplex of two stretches of 13 base pairs separated by a bulged loop that sequestered the RBS, and a loop-loop interaction that took place in the coding region. In contrast to *rot* mRNA, in which the two loop-loop interactions were essential for *in vivo* repression, the sequestration of the RBS of *coa* mRNA is sufficient by itself to promote efficient repression *in vivo* and to prevent the formation of the ribosomal initiation complex (Fig. 5A, 6). Indeed, the loop-loop interaction is not essential for efficient *in vivo* repression and contributes only moderately to the stability of the inhibitory complex. Hence, the various topologies of the repressed RNAIII-mRNA complexes depend largely on the mRNA context.

The inhibitory RNAIII-*coa* mRNA complex also provided specific binding sites for the double strand-specific RNase III, which induced strong cleavages in the two regions of *coa* mRNA bound to RNAIII. Notably, the cleavage sites in the loop-loop interaction also occurred at a similar position in the two kissing interactions that took place in the *rot* mRNA-RNAIII complex (Fig. S3; [12]). The sequences of *coa* mRNA involved in the loop-loop interactions are very similar to *rot* mRNA, showing that similar signatures exist in various RNAIII-repressed mRNAs (Fig. S3). Molecular modeling of the kissing interaction, which took into account the chemical and enzymatic probing data, revealed that the loop-loop interaction induces a coaxial stacking of the two intramolecular helices (Fig. 3C). The overall topology is very similar to the RNA loop-loop structure obtained by NMR, which mimics the interaction between sense and antisense RNAs involved in the regulation of the ColE1 plasmid [26]. Such a long helical structure might well be appropriate for the binding of the homodimeric enzyme, although the sequence of the kissing interactions might also be a specific binding determinant. Of interest, *coa* mRNA was shown to be completely depleted as soon as RNAIII was produced, and the deletion of *rnc* caused the accumulation of *coa* mRNA ([13]; Fig. S2). Therefore, as we postulated previously, RNase III might initiate rapid degradation of *coa* mRNA, and the cleavage in the loop-loop interaction may also contribute to access to several other endo- or exoribonucleases for further degradation (Fig. 6). Notably, at a similar position, *spa* and SA1000 mRNAs carry a long stem-loop structure in the coding sequence that is also cleaved efficiently by RNase III [11],[12] (Fig. S3). In addition, the depletion of the mRNA might also result from an indirect effect of RNAIII. Indeed, Rot protein was shown to activate the transcription of *coa* mRNA [27], while RNAIII represses the synthesis of Rot at the post-transcriptional level [10]. Thus, the RNAIII-mediated repression of coagulase would occur at both transcriptional and post-transcriptional levels as it was shown for *spa* mRNA [10],[12],[27].

It is not an exception that RNAIII utilizes conserved C-rich loops to target similar regions of various mRNAs that are functionally related. In *S. typhimurium*, GcvB RNA represses translation initiation of multiple target mRNAs by binding to a C/A-rich motif present in all these mRNAs, which encode periplasmic substrate-binding proteins of ABC uptake systems for amino acids and peptides [28],[29]. *E. coli* CyaR contains a hairpin loop with a conserved anti-SD sequence that is used to target the SD sequence of a subset of mRNAs [30],[31]. Similarly to RNAIII, we recently found that other *S. aureus* ncRNAs carry a similar UCCC signature always present in an unpaired region, and through its unpaired C-rich motif, one of these RNAs binds to the RBS and represses the expression of several mRNAs [32]. *S. aureus coa* mRNA and the other mRNA targets of RNAIII carry a strong SD sequence located in an unpaired region that is quite appropriate for the docking of the 30S subunit, but also for the formation of initial contacts with the C-rich loop of RNAIII (Fig. S1). Specificity for *coa* regulation is mainly given by the propagation of the intermolecular contacts to form a long imperfect duplex further stabilized by a loop-loop interaction in the coding sequence.

In conclusion, this study validates another direct target of RNAIII that plays a role in virulence. Our study further stresses that the RNAIII harbors highly conserved regions that provide a specific signature to generate interactions with the RBS of multiple mRNAs and that the mRNA context directs the topology of the inhibitory complexes. Recent works focusing on *E. coli* and *S. typhimurium* show that regulatory RNAs that target mRNAs regulate gene expression through a variety of unusual mechanisms and bind to mRNA regions located far away from the ribosome binding site in the 5′UTR [33], in the coding sequence [34],[35], and in the 3′ end [36]. Whether *S. aureus* has also evolved such a diversity of RNA-dependent regulatory mechanisms remains to be addressed.

## Materials and Methods

### Strains and plasmids

*S. aureus* RN6390 or LUG1467 derives from 8325-4. In WA400 and LUG1457 (Δ*rnaIII*), the P3 operon is deleted and replaced by the chloramphenicol transacetylase gene (*cat86*) [37]. LUG774 and LUG911 strains derive from RN6390, in which *rnc* and *hfq* genes, respectively, have been replaced by *cat86* gene [11]. Staphylococci were grown either on BM agar plates (1% peptone, 0.5% yeast extract, 0.1% glucose, 0.5% NaCl, 0.1% K_2_HPO_4_) or in brain-heart infusion (BHI) with erythromycin (5 µg/ml) when appropriate. RNAIII and its variants were expressed in *Staphylococcus aureus* WA400 with plasmid pE194 (see Table 1).

**Table 1:** Strains and plasmids.

*S. aureus* strains	Relevant characteristics	Reference or source
RN4220	restriction- mutant of 8325-4	[45]
RN6390	derivative of 8325-4, *agr* positive	[46]
WA400	8325-4: Δ*rnaIII* region::*cat*86	[37]
LUG 404	W400/pLUG 274	[12]
LUG 450	WA400/pLUG 300	[11]
LUG 453	WA400/pLUG 304	[11]
LUG 580	WA400/pLUG 394	this study
LUG 581	W400/pLUG 298	[11]
LUG774	RN6390: Δ*rnc* region::*cat*86	[11]
LUG911	RN6390: Δ*hfq* region::*cat*86	[12]
LUG 1445	LUG 911/pLUG 745	this study
LUG 1446	LUG 774/pLUG 745	this study
LUG 1447	LUG 450/pLUG 745	this study
LUG 1454	LUG 453/pLUG 745	this study
LUG 1456	LUG 580/pLUG 745	this study
LUG 1457	WA400/pLUG 745	this study
LUG 1467	RN6390/pLUG 745	this study
LUG 1474	LUG 581/pLUG 745	this study
LUG 1478	LUG 404/pLUG 745	this study
***E.coli*** **-staphylococcal shuttle plasmids**		
pTCV-*lac*	Promoter-lac fusion shuttle vector: *spoVG-lacZ*, *ermB*, *aphA-3*	[14]
pLUG 220	pTCV-*lac* ΔRBS and start codon	[11]
pLUG 745	pLUG220::P*rpo*B (nts −480 to +1)::*coa* (+1 [transcriptional start] to +126)	this study
**Staphylococcal plasmids**		
pE194	3.728 kb *S. aureus* plasmid, inducible MLS resistance (*erm*)	[47]
pLUG 274	pE194::EcoRV site in MCS	[38]
pLUG 298	pLUG274::P3 operon (nts 1819–751)	[11]
pLUG 300	pLUG274::P3 promoter link to 3′ domain of RNAIII (nts 391–514)	[38]
pLUG 304	pLUG274::*rnaIII* Δnts 408–451 (RNAIII-Δ13)	[11]
pLUG 394	pLUG274::*rnaIII* Δnts 208–323 (RNAIII-Δ7-9)	this study

### Construction of translation fusions and β-galactosidase measurements

Translation fusions were constructed with plasmid pLUG220, a derivative of pTCV-*lac*, a low-copy-number promoter-less *lacZ* vector (Table 1). The 5′ end of the *coa* mRNA was first determined by rapid amplification of cDNA ends (RACE) using the First Choice RLM-RACE kit following the company's protocol (Ambion). The whole leader region of *coa* mRNA including 126 nt of the coding sequence, was cloned downstream the *rpo*B promoter in frame with *lac*Z [11]. β-galactosidase activity was measured three times on duplicate cultures with the Enzyme Assay System (Promega).

### Northern blots and measure of mRNA half-life

Electrophoresis of total RNA (20 µg) was done on a 1% agarose gel containing 2.2 M formaldehyde and vacuum transfer to nylon membrane. Hybridizations with specific digoxigenin-labeled RNA probes complementary to *coa* mRNA and luminescent detection were carried out as described previously [13].

### RNA preparation

RNAIII, RNAIII derivatives (RNAIII-Δ7–9: deletion of nts G207 to U319, RNAIII-Δ13: deletion of nts U409 to A451, and RNAIII-Δ14: deletion of nts G483 to C511, the 3′ domain comprises nts 391 to 516), the isolated hairpin 7, and the *coa* mRNA fragment were transcribed *in vitro* using T7 RNA polymerase as described previously [38]. The transcribed RNAs were purified by 8% polyacrylamide-8 M urea gel electrophoresis. After elution in 0.5 M ammonium acetate/1 mM EDTA buffer, the RNAs were precipitated twice with ethanol. Before use, the pellet was dissolved in sterile bi-distillated water and the concentration was measured accurately.

The 5′ end-labeling of dephosphorylated RNA or DNA oligonucleotides was performed with T4 polynucleotide kinase and [γ-^32^P]ATP [39]. Before use, RNAs were renatured by incubation at 90°C for 2 min in the absence of magnesium and salt, 1 min on ice, followed by an incubation step at 20°C for 15 min in TMN buffer (20 mM Tris-acetate pH 7.5, 10 mM magnesium-acetate, 150 mM Na-acetate).

### Determination of constants of RNAIII-*coa* mRNA complex formation

Binding rate constant of RNAIII-*coa* mRNA complex was measured as described previously [40]. Binding of end-labeled *coa* mRNA to a ten-fold excess of unlabeled RNA (RNAIII, RNAIII-Δ13, RNAIII-Δ7-9) was performed at 37°C in TMN buffer. Samples were withdrawn at various time points (0–10 min), added to gel application buffer and loaded onto a native 5% polyacrylamide gel. The gel was run at 4°C and constant voltage (300 V) for 3 h and subsequently dried. Bands corresponding to the RNAIII-*coa* mRNA complex and free RNAIII, respectively, were quantified using the SAFA algorithm [41].

For determination of the dissociation rate constant of RNAIII-*coa* mRNA complex, end-labeled *coa* mRNA was incubated with an increased molar amount of wild-type RNAIII or RNAIII variants (RNAIII-Δ13, RNAIII-Δ7-9, 3′ domain, hairpin 7) for 15 min at 37°C in TMN buffer. Samples were then treated as described above. All experiments were done four times giving reproducible data.

### RNA structure probing

RNAIII-*coa* mRNA formation was carried out at 37°C for 15 min in TMN buffer. Enzymatic hydrolysis was performed in 10 µl of TMN, in the presence of 1 µg carrier tRNA at 37°C for 5 min: RNase T1 (0.0025 units), RNase V1 (0.5 units). Chemical modifications were performed on 2 pmol of *coa* mRNA or RNAIII at 20°C in 20 µl of reaction buffer containing 2 µg of carrier tRNA. Alkylation of C(N3) and A(N1) positions was done with 1 µl DMS (diluted 1/8 and 1/16 in ethanol) for 2 min, and modification of A(N7) was done with 4 µl of DEPC for 20 min at 20°C in TMN buffer. Modifications of U(N3) and G(N1) were done with 5 µl of CMCT (50 mg/ml) for 10 and 20 min in a buffer containing 50 mM Na-borate pH 8, 5 mM MgAc, and 150 mM KOAc. RNase III purification and the enzymatic cleavage assays on *coa* mRNA and on RNAIII were performed as described previously [18].

End-labeled RNA fragments were sized on 12% polyacrylamide/8 M urea slab gels. Cleavage positions were identified using RNase T1 and alkaline ladders of the probed RNA. The cleavage or modification sites of unlabeled RNAs were detected by primer extension. Details for hybridization conditions, primer extension, and analysis of the data have been described previously [38].

### Toeprinting assays

*S. aureus* 30S subunits were prepared according to [42]. The formation of a simplified translational initiation complex with mRNA and the extension inhibition conditions were strictly identical to those described by [38],[42]. Standard conditions contained 15 nM *coa* mRNA annealed to a 5′ end-labeled oligonucleotide complementary to nts 99 to 117 of *coa* mRNA, 250 nM *S. aureus* 30S ribosomal subunits (250 nM), and 25 to 100 nM of RNAIII or its variants in 10 µl of buffer containing 20 mM Tris-acetate, PH 7.5, 60 mM NH4Cl, 10 mM magnesium acetate, and 3 mM β-mercaptoethanol. After 10 min at 37°C, the initiator tRNA (1 µM) was added and the reaction was incubated for a further 5 min at 37°C. Reverse transcription was conducted with one unit of AMV reverse transcriptase for 15 min at 37°C. Relative toeprinting (toeprint band over full-length RNA+toeprint) was calculated by scanning of the gel with the Bio-imager Analyser (Fuji).

### Molecular modeling

Modeling of the regions encompassing residues U73 to A114 of the *coa* mRNA and of residues A223 to U256 of RNAIII were carried out as described [43],[44]. Following the interactive assembly step, several cycles of geometrical least-square refinements were performed until a satisfactory solution was reached. Figure 3C was prepared using the PYMOL program (DeLano WL, The PyMOL Molecular Graphics System 2002; http://www.pymol.org).

## Supporting Information

Figure S1 Secondary structure of *Staphylococcus aureus* RNAIII. The 3′ domain is squared. Arrows indicate the deletions of the hairpin 13 (RNAIII-Δ13), or of the three hairpins 7 to 9 (RNAIII-Δ7–9). The hairpin 7 used in the RNase III hydrolysis is shown. The secondary structure of RNAIII was experimentally defined [38]. The drawing is adapted from Figure 1A in Boisset et al. [12] (copyright permission from *Genes & Development*, Cold Spring Harbor Laboratory Press, USA).
(0.87 MB EPS)

Figure S2 Northern blot analysis on *coa* mRNA prepared from exponential phase cultures (OD_600nm_ 0.5) from various *S. aureus* strains. The blot was hybridized with an RNA probe antisense to *coa* mRNA: RN6390 (WT, *rnaIII*+); *S. aureus* strain lacking *rnaIII* gene, WA400 (Δ*rnaIII*); *S. aureus* strain lacking *rnc* gene, Δ*rnc* strain (LUG774); WA400 transformed with a plasmid expressing the 3′ domain of RNAIII (Δ*rnaIII* + 3′ Dom) or the RNAIII deleted of hairpin 13 (Δ*rnaIII* + RNAIII-Δ13). Ribosomal RNAs were visualized on the same membrane by ethidium bromide staining, as an internal control (data not shown). Three independent experiments provided reproducible results.
(3.85 MB TIF)

Figure S3 Comparison of the secondary structures of the RNAIII targets. (A) The secondary structures of *spa* mRNA [11], SA1000 and *rot* mRNA [12], and *coa* mRNA. The nucleotides base-paired to RNAIII are in red. The conserved G-rich sequence in the mRNAs, which interact with the C-rich motif of hairpin loops 7, 13, 14 of RNAIII are encircled in pink. (B) Sequences of the loop-loop interactions as found in *rot* mRNA-RNAIII and *coa* mRNA-RNAIII complexes. Arrows denote the RNase III cleavages.
(0.47 MB EPS)
