# Supplementary figures and images for: Staphylococcus aureus RNAIII Binds to Two Distant Regions of coa mRNA to Arrest Translation and Promote mRNA Degradation
Source: PLoS Pathog. 2010 Mar 12;6(3):e1000809. doi: 10.1371/journal.ppat.1000809 (PMC2837412; doi:10.1371/journal.ppat.1000809)

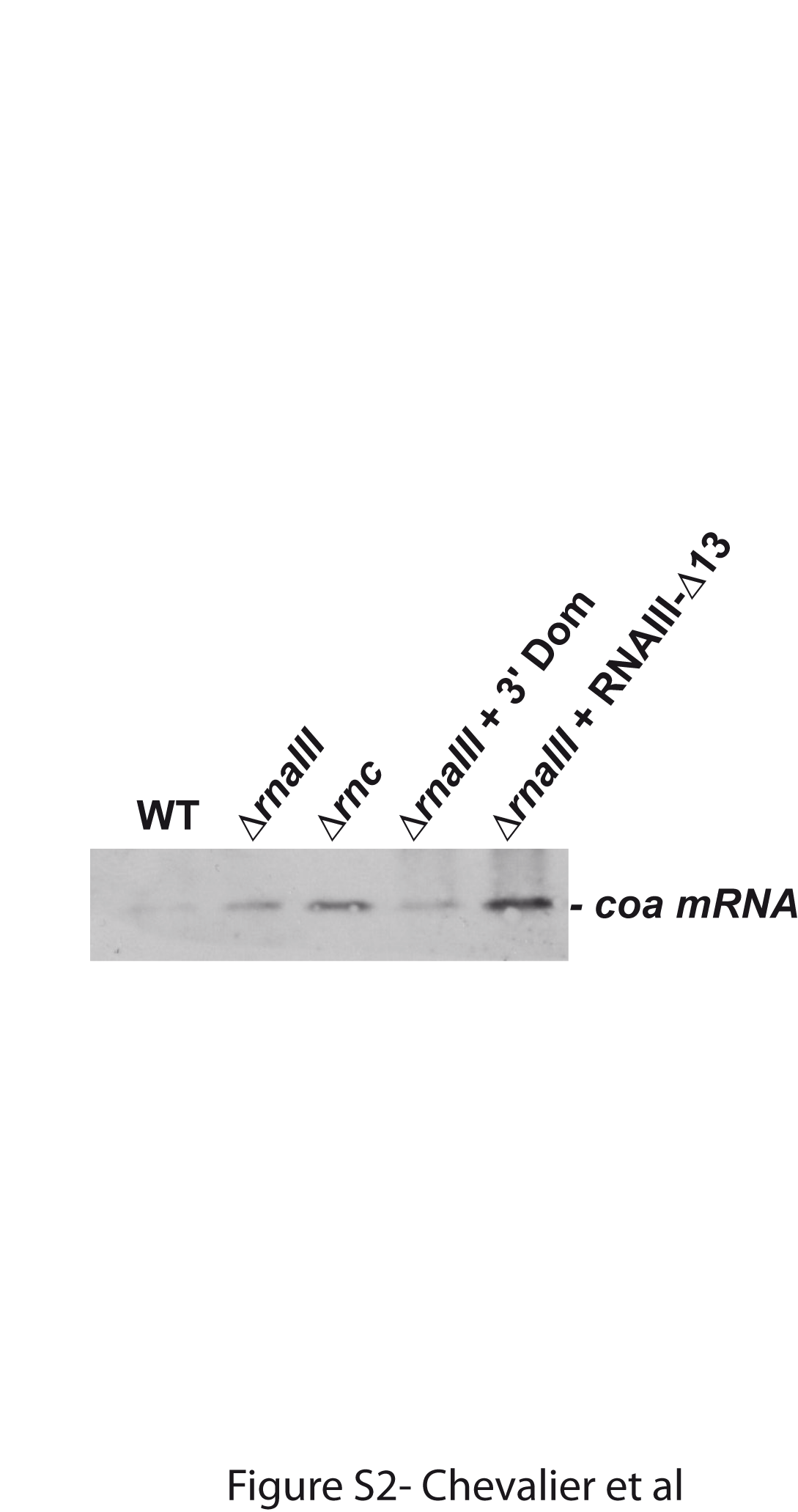

Supplement: Figure S2 — Northern blot analysis on coa mRNA prepared from exponential phase cultures (OD600nm 0.5) from various S. aureus strains. The blot was hybridized with an RNA probe antisense to coa mRNA: RN6390 (WT, rnaIII+); S. aureus strain lacking rnaIII gene, WA400 (ΔrnaIII); S. aureus strain lacking rnc gene, Δrnc strain (LUG774); WA400 transformed with a plasmid expressing the 3′ domain of RNAIII (ΔrnaIII + 3′ Dom) or the RNAIII deleted of hairpin 13 (ΔrnaIII + RNAIII-Δ13). Ribosomal RNAs were visualized on the same membrane by ethidium bromide staining, as an internal control (data not shown). Three independent experiments provided reproducible results. (3.85 MB TIF) [file ppat.1000809.s002.tif]
